# Immunomodulatory role of vitamin D and selenium supplementation in newly diagnosed Graves’ disease patients during methimazole treatment

**DOI:** 10.3389/fendo.2023.1145811

**Published:** 2023-04-14

**Authors:** Daniela Gallo, Antonino Bruno, Matteo Gallazzi, Simona Antonia Maria Cattaneo, Giovanni Veronesi, Angelo Genoni, Maria Laura Tanda, Luigi Bartalena, Alberto Passi, Eliana Piantanida, Lorenzo Mortara

**Affiliations:** ^1^ Endocrine Unit, Department of Medicine and Surgery, University of Insubria, Azienda Socio-Sanitaria Territoriale dei Sette Laghi, Varese, Italy; ^2^ Immunology and General Pathology Laboratory, Department of Biotechnology and Life Sciences, University of Insubria, Varese, Italy; ^3^ Laboratory of Innate Immunity, Unit of Molecular Pathology, Biochemistry, and Immunology, Istituto di Ricovero e Cura a Carattere Scientifico (IRCCS) MultiMedica, Milan, Italy; ^4^ Immuno-hematology and Transfusion Medicine, Azienda Socio-Sanitaria Territoriale dei Sette Laghi, Varese, Italy; ^5^ Research Centre in Epidemiology and Preventive Medicine (EPIMED), Department of Medicine and Surgery, University of Insubria, Varese, Italy; ^6^ Azienda Socio-Sanitaria Territoriale dei Sette Laghi, Department of Medicine and Surgery, University of Insubria, Varese, Italy; ^7^ Laboratory of Clinical Chemical Analysis, Department of Medicine and Surgery, University of Insubria, Azienda Socio-Sanitaria dei Sette Laghi, Varese, Italy

**Keywords:** Graves‘ disease, vitamin D, selenium, natural killer (NK) cell, T regulatory cells (T reg)

## Abstract

**Introduction:**

Methimazole (MMI) represents the conventional therapeutic agent for Graves’ disease (GD) hyperthyroidism, but MMI efficacy is limited since it marginally affects the underlying autoimmune process. In a previous study, we randomly assigned 42 newly diagnosed GD patients with insufficient vitamin D (VitD) and selenium (Se) levels to treatment with MMI alone (standard) or combined with selenomethionine and cholecalciferol (intervention) and observed a prompter resolution of hyperthyroidism in the intervention group.

**Methods:**

In the present study, we aimed to explore changes in peripheral T regulatory (Treg) and circulating natural killer (NK) cell frequency, circulating NK cell subset distribution and function, during treatment.

**Results:**

At baseline, circulating total CD3^-^CD56^+^NK cells and CD56^bright^ NK cells were significantly higher in GD patients than in healthy controls (HC) (15.7 ± 9.6% vs 9.9 ± 5.6%, p=0.001; 12.2 ± 10.3% vs 7.3 ± 4.1%, p=0.02, respectively); no differences emerged in Treg cell frequency. Frequencies of total NK cells and CD56^bright^ NK cells expressing the activation marker CD69 were significantly higher in GD patients than in HC, while total NK cells and CD56^dim^ NK cells expressing CD161 (inhibitory receptor) were significantly lower. When co-cultured with the K562 target cell, NK cells from GD patients had a significantly lower degranulation ability compared to HC (p<0.001). Following 6 months of treatment, NK cells decreased in both the intervention and MMI-alone groups, but significantly more in the intervention group (total NK: -10.3%, CI 95% -15.8; -4.8% vs -3.6%, CI 95% -9; 1.8%, p=0.09 and CD56^bright^ NK cells: -6.5%, CI 95% -10.1; -3 vs -0.9%, CI 95% -4.4; 2%, p=0.03). Compared to baseline, CD69^+^ NK cells significantly decreased, while degranulation ability slightly improved, although no differences emerged between the two treatment groups. Compared to baseline, Treg cell frequency increased exclusively in the intervention group (+1.1%, CI 95% 0.4; 1.7%).

**Discussion:**

This pilot study suggested that VitD and Se supplementation, in GD patients receiving MMI treatment, modulates Treg and NK cell frequency, favoring a more pronounced reduction of NK cells and the increase of Treg cells, compared to MMI alone. Even if further studies are needed, it is possible to speculate that this immunomodulatory action might have facilitated the prompter and better control of hyperthyroidism in the supplemented group observed in the previous study.

## Introduction

Graves’ disease (GD) is a common autoimmune disorder (1), in which thyrotropin-receptor antibodies (TSHR-Ab) inappropriately stimulate thyrocytes *via* the TSH-R to uptake iodine, proliferate and synthesize excess thyroid hormones (2). Clinically, this interaction causes hyperthyroidism and goiter, and may be accompanied by extrathyroidal manifestations, the most common being GD orbitopathy (GO) (1–5). Thionamide antithyroid drugs (ATD), the common first-line treatment for GD, inhibit thyroid hormone synthesis but have little, if any, direct effect on the immune dysregulation responsible for the disease, and are, therefore, bound to a high relapse rate (4). A key and unmet goal of research is to identify pharmacological treatments targeting the immunological mechanisms responsible for the disease (5).

GD develops because of the abnormal activation of T and B cells, which ultimately leads to the production of TSHR-Ab and other mediators, such as chemokines and cytokines, involved in autoimmune inflammation (6). In normal individuals, T regulatory (Treg) cells are engaged in keeping autoreactive immune cells under control, thus preventing the development of autoimmune diseases. Either a low number or defective suppressive activity of Treg cells has been reported in GD (7, 8). Natural killer (NK) cells, and particularly CD56^bright^CD16^low^NK cells, also uphold immune-regulatory actions (9, 10). NK cells quickly recognize and kill stressed cells because of infection or malignancy by integrating signals from inhibitory and activating receptors and orchestrate the innate and adaptive immune responses by producing various cytokines and chemokines or *via* the crosstalk with other immune cells (9). NK cell involvement in GD is still a matter of argument, although the few available studies mostly agree on decreased degranulation and cytokine release in GD patients compared to healthy subjects (10).

We recently reported, in a randomized control trial, that in newly diagnosed GD patients the association of cholecalciferol (VitD) and selenium (Se) supplementation with methimazole (MMI), in patients with low VitD and selenium levels, significantly accelerated the control of hyperthyroidism, during the first 6 months of treatment, compared to MMI alone (11). We postulated that this might be, at least partly, due to the influence of micronutrients on the immune response, as suggested by animal and cell system-based studies (12–20).

To address this issue, we investigated the effect of the association of selenium and cholecalciferol supplementation with standard MMI treatment on circulating Treg frequency and circulating NK cells frequency, NK cells phenotype and functions in GD patients enrolled in our previous study (11).

## Materials and methods

### Design, setting, treatment and participants

**Figure 1:**
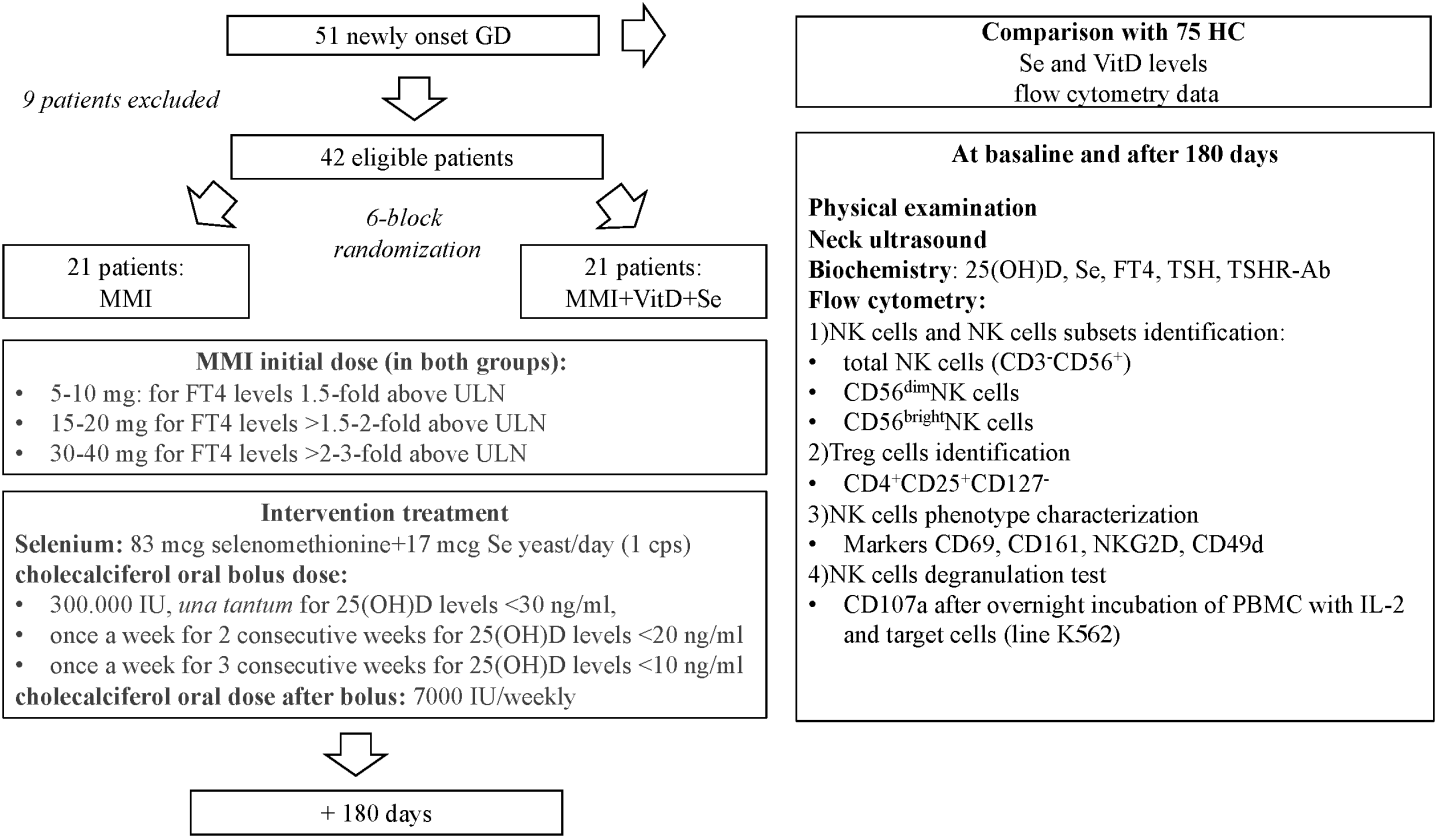
Flow-chart describing the design of the study. GD, Graves' disease patients; HC, healthy controls; IL, interleukin; MMI, methimazole; NK, natural killer cells; PBMC, peripheral blood mononuclear cells; Se, selenium; Treg, T regulatory cells; TSHR-Ab, TSH receptor antibodies; ULN, upper limit of normal; VitD, vitamin D levels; 25(OH)D, calcidiol.

This was a randomized, single-blinded, monocentric, controlled, intervention trial (registration number on Eudract Clinical Trial Register SeMMIviD 2017-005050-11) (11). Study protocol, inclusion and exclusion criteria are summarized in **Figure 1**. Newly diagnosed GD patients, with marginally low circulating calcifediol (< 30 ng/ml) and Se (< 120 mcg/liter) levels were consecutively recruited at the Endocrine Unit (Varese) and randomized to treatment with MMI monotherapy (standard treatment) or MMI combined with Se and VitD (intervention treatment) (11). All GD patients were treated with MMI, at doses based on free thyroxine (FT4) levels assessed at baseline and after 30, 45, and 180 days of treatment and modulated according to the “titration methods” as per our clinical practice (**Figure 1**). The intervention group was supplied with Se 100 mcg/day (selenomethionine 83 mcg + selenium yeast 17 mcg) and with cholecalciferol (oral bolus dose calculated on baseline 25(OH)D levels followed by 7000 UI/weekly for the trial duration) (**Figure 1**) (11). Adequate supplies of Se and cholecalciferol were provided at each study appointment to ensure compliance.

Peripheral blood samples for immunological studies were collected at baseline and after 180 days.

Seventy-five age- and sex-matched euthyroid healthy controls (HC), without thyroid autoantibodies and not taking dietary supplements, were consecutively enrolled among volunteers attending the Transfusion Medicine Centre in Varese.

Written informed consent was collected at enrolment from all participants and personal details were anonymized. The study was approved by the local Ethics Committee (ASST dei Sette Laghi, Varese, registration number 92/2017) and by Agenzia Italiana del Farmaco (measure SC 14282).

### Study outcomes

The primary outcome was the between-arms (standard MMI alone vs intervention treatment) difference in the variation of circulating Treg and NK cell frequencies from baseline to 180 days. Secondary outcomes included the study of NK cell cytotoxicity and phenotypic markers at baseline and after 180 days of treatment.

## Methods

### Flow cytometry

Immunophenotyping for circulating T cell and NK cell subsets was performed by multicolour flow cytometry (Becton Dickinson Biosciences FACS Canto II Instrument equipped with 3 laser, San Josè California, USA), after staining total mononuclear cells (MNCs) with fluorophore-conjugated monoclonal antibodies (mAbs, BD Biosciences or Miltenyi Biotech). Flow data were analyzed by BD FACS DivaTM software (Becton Dickinson Biosciences, SAN Josè California, USA). Treg cells were selected on CD45^+^ (Horizon V500-conjugated anti-CD45, clone 2D1), CD4^+^ (Horizon V-450- conjugated anti-CD4, clone SK3), CD3^+^ (FITC-conjugated anti-CD3, clone OKT3), CD8^-^ (APC-H7-conjugated anti-CD8, clone SK1) gated lymphocytes, and further identified as CD25^+^ (PE-conjugated anti-CD25, clone 2A3), CD127^low^ (APC-conjugated anti-CD127, clone A019D5) cells. Total NK cells were identified as CD45^+^ as apex (Horizon V500-conjugated anti-CD45, clone 2D1), CD3^-^ (PerCP-conjugated anti-CD3, clone SK7), CD56^+^ (APC-conjugated anti-CD56 (NCAM), clone HCD56) cells and then assessed as CD56^bright^ and CD56^dim^. The following PE-conjugated antibodies were used to detect the percentage of total NK and NK CD56^bright^ and CD56^dim^ subsets expressing the stimulatory receptor CD69 (clone FM50), activation receptor NKGD2 (CD314, clone 1D11), inhibitory receptor and CD161 (clone HP3G10), and the α4 integrin CD49d (clone 9F10).

### Degranulation assay

Total peripheral blood mononuclear cells (PBMC) were co-cultured with the human immortalized myelogenous leukemia line K562 cells (1:1 ratio) (ATCC), incubated with FITC-conjugated anti-CD107a mAb for 4 hours (37°C, 5% CO_2_). Degranulation capability of NK cells (total, CD56^bright^ and CD56^dim^ subsets) was determined by flow cytometry detection of CD107a production (BD FACS Canto II Instrument, BD Biosciences); flow data were analyzed using the BD FACS DivaTM and the FlowJo v10 softwares.

### Biochemistry

Serum TSH (reference range: 0.27-4.2 mU/liter), FT4 (9.3-17 pg/ml) and FT3 (FT3, 2-4.4 pg/ml) concentrations were measured by electrochemiluminescence immunoassay (Analytical Unit for immunochemistry Cobas e801, Roche), TSHR-Ab title (normal value: <1 U/liter; upper limit of detection: 40 U/liter) by second-generation radioreceptor assay (Thermofisher, Germany). Serum Se levels (limit of detection 10 mcg/liter, inter-day variation coefficient 4.3%) were measured by a transversely heated graphite atomizer furnace atomic absorption spectrometer (Analyst 600, PerkinElmer^®^, Waltham, USA) equipped with an electrodeless discharge lamp. Liquid chromatography/mass spectrometry (Shimadzu^®^ Nexera Liquid Chromatography modules coupled to Sciex^®^ Triple Quad 6500^+^ Mass Spectrometer) was used for plasma ViD assay (intraassay variation coefficient 3.2%, interassay variation coefficient 3.4%).

### Statistical analyses

Statistical analyses were performed using the SAS software 9.4 release. The null hypothesis of no difference in time change between the study groups was tested using a Wald chi-square test with 4 degrees of freedom. Furthermore, we report the p-values for cross-sectional comparisons in NK cells and Treg cell frequencies between groups, at baseline and after 180 days. Continuous data were reported as mean ± standard deviation (SD) or standard error of the mean (SEM). Discrete variables were reported as percentages.

## Results

**Figure 2:**
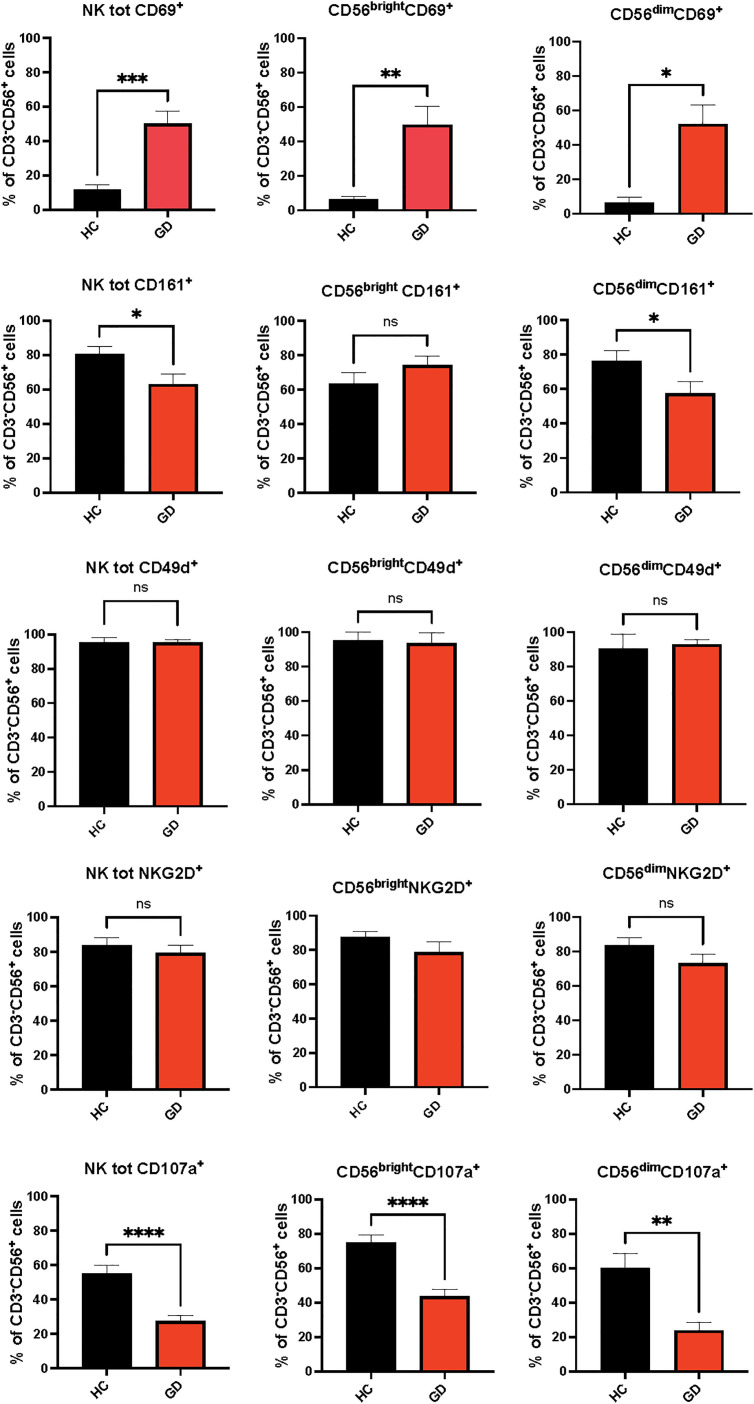
Phenotypic (CD69, CD161, NKG2D, CD49d) and functional (CD107a) results of natural killer cells from Graves’ disease patients, at baseline, and healthy controls. Twenty-five age and sex-matched healthy donors served as control group. Histograms showed the percentages of NK cells total NK cells and CD56bright and CD56dim subsets expressing activating (CD69+), inhibitory (CD161^+^, NKG2D^+^) receptors, the integrin CD49d and having cytotoxic ability (CD107a^+^). Data were shown as mean SEM (standard error of the mean), student t-test, *p<0.05, **p<0.01, ***p< 0.001,****p<0.0001. GD, Graves’ disease patients; HC, healthy controls; MMI, methimazole. ns, not statistically significant.

Fifty-one newly diagnosed GD patients were assessed for VitD and Se levels, which were significantly lower when compared to HC (respectively, VitD levels: 19 ± 27.3 ng/ml vs 29.5 ± 11.7 ng/ml, p<0.001; Se levels: 93.7 ± 15.8 mcg/l vs 107.4 ± 12.2 mcg/l, p<0.05) (data not shown). After excluding 9 patients having normal VitD and/or Se levels or taking supplements, 42 patients were included (37 women and 5 men, aged 45.8 ± 10.3 years) (**Table 1**). Compared to HC, GD patients had higher total NK cells (p-value t-test for independent sample 9.9 ± 5.6% vs 15.7 ± 9.6%, p 0.001) and CD56^bright^NK cells (7.3 ± 4.1% vs 12.2 ± 10.3%, p=0.02) (**Supplementary Table 1**). The proportion of circulating Treg cells (6.8 ± 1.7% vs 7.5 ± 2%, p=0.11) was not significantly different comparing GD patients and HC (**Supplementary Table 1**). Age was inversely related to the frequency of CD56^bright^NK cells (r=-0.346, p<0.05) and directly related to Treg cell frequency (r=0.554, p<0.05) (**Supplementary Table 2**). As shown in **Figure 2**, the frequencies of total NK cells and CD56^bright^ NK cells expressing the activating receptor CD69 were significantly higher in GD patients compared to HC, while total NK cells and CD56^dim^NK cells expressing the inhibitory marker CD161 were significantly lower in GD patients than in HC. Although the percentage of NK cells expressing the activating receptor NKG2D (62.7 ± 21.4% vs 93 ± 3.2%, p>0.05) was lower in GD patients compared to HC, this trend was not significant. There was no statistically significant difference in the frequency of NK cells expressing the integrin CD49d (92.212% vs 97.19%, p>0.05). More in-depth functional studies showed that total NK cells (mean ± SEM, 55.01 ± 27.55 vs 27.46 ± 5.72, p<0.0001), CD56^bright^NK cells (75.04 ± 31.26 vs 43.08 ± 5.89, p<0.0001) and CD56^dim^ (59.98 ± 35.99 vs 23.98 ± 9.11, p<0.001) NK cell subsets from GD patients had significantly lower degranulation ability compared to HC (**Figure 2**). As detailed previously (11) and summarized in **Table 1**, all GD patients had overt hyperthyroidism and VitD insufficiency but GD patients in the intervention treatment group had significantly higher FT4 levels and significantly lower VitD levels compared to the standard treatment group. Pearson correlation test did not find a significant correlation between baseline VitD, Se levels, NK and Treg cells frequencies (**Supplementary Table 2**) at baseline but detected a negative correlation between VitD levels and the frequency of NK cells (total) expressing CD161 (r=-0.707, p<0.005), (data not shown).

**Table 1:** Baseline main demographic, clinical and biochemical features of 42 GD patients (11).

Parameters	Whole sample	MMI	MMI+Se+VitD	p
***Number***	**42**	**21**	**21**	**-**
***Age, years***	45.8 ± 10.3	47.7 ± 11.4	45.8 ± 9.3	0.55
***Sex, n. women (%)***	36 (87.8)	19 (95)	17 (81)	0.17
***Se, mcg/l***	93 ± 10.2	94.9 ± 10.9	91.2 ± 9.4	0.25
***VitD, ng/ml***	19.2 ± 7.3	22.9 ± 6.3	15.7 ± 6.5	**0.001**
***Treg, %***	7.4 ± 2	7.8 ± 2.4	7.1 ± 2	0.37
***NK, %***	15.7 ± 9.6	11 ± 7.4	16.6 ± 11.9	0.10
***CD56^bright^NK, %***	12.2 ± 10.3	12.9 ± 8.5	16.5 ± 11.6	0.30
***CD56^dim^NK, %***	84.5 ± 11.5	82.6 ± 10.4	80.7 ± 13.4	0.65

Se, selenium; VitD, vitamin D; Treg, T regulatory cells; n., number; NK, natural killer cells; %, frequency. Data were reported as mean ± standard deviation or frequency (percentage). p=p value testing baseline differences between the standard treatment group (MMI=methimazole) and the intervention treatment group (MMI+Se+VitD). Bold values idientified p<0.05. Bold values for p values means statistically significant difference.

After 6 months, VitD and Se levels significantly increased in the intervention group, as proof of adequate adherence to supplementation. Data on thyroid function variation are beside the topic of this study and were detailed previously (11). Briefly, after 6 months of treatment, FT4 levels had a significantly higher decrease from baseline in the intervention group compared to MMI monotherapy group (respectively -36.5 pg/ml, CI 95%, -42.1 to -30.8 pg/ml vs -25.7 pg/ml, CI 95%, -32.3 to -19 pg/ml and -22.9 pg/ml, CI 95%, -28.7 to -17.2 pg/ml, p 0.03); thus, mean FT4 values at 45 and 180 days were similar comparing the two groups (10).

**Figure 3:**
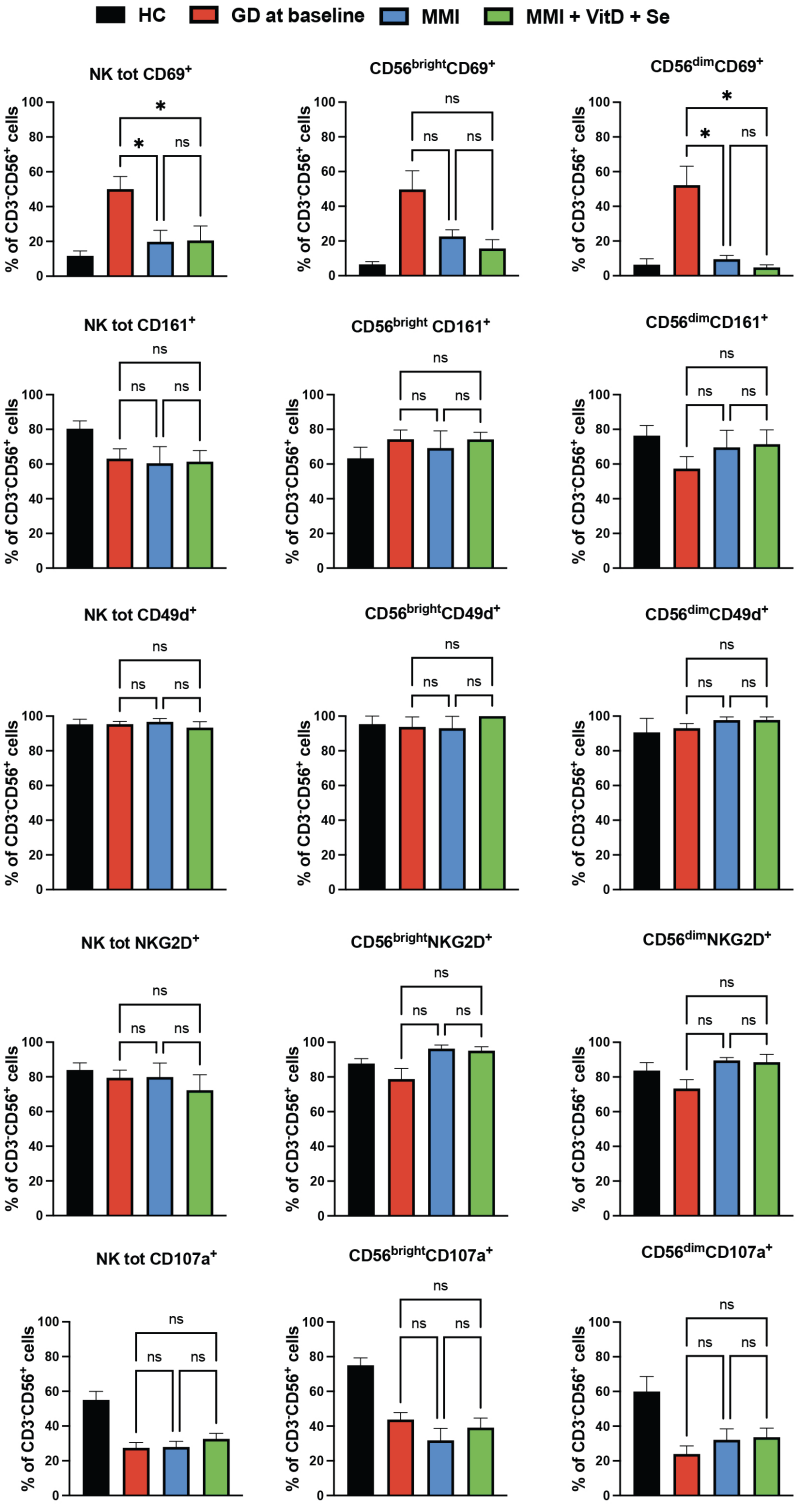
Phenotypic (CD69, CD161, NKG2D, CD49d) and functional (CD107a) data of natural killer cells from Graves’ disease patients, at baseline, and after six months of treatment, divided by group of intervention Twenty-five age and sex healthy donors served as control group. Histograms showed the percentages of NK cells expressing activating (CD69+), inhibitory (CD161+, NKG2D+) receptors, the integrin CD49d and having cytotoxic ability (CD107a+). Data were shown as mean SEM (standard error of the mean), student t-test, *p<0.05. GD, Graves’ disease patients; MMI, methimazole. NS, not statistically significant.

Total NK and NK cells subsets significantly decreased in both groups, but changes in total NK and in CD56^bright^NK cells were significantly higher in the intervention group compared to standard monotherapy (intervention group vs standard MMI, total NK: -10.3%, CI 95% -15.8; -4.8% vs -3.6%, CI 95% -9; 1.8% vs p 0.09 and CD56^bright^NK cells: -6.5%, CI 95% -10.1; -3 vs -0.9%, CI 95% -4.4; 2%, p=0.03) (**Table 2**). Compared to the baseline, the percentage of total CD69^+^NK cells and of CD69^+^CD56^dim^NK cells significantly decreased, while degranulation slightly increased in total NK cells and CD56^dim^NK cells in both groups (**Figure 3**). Circulating Treg cells followed an opposite trend in the two groups of treatment (interaction between time course and treatment group, p=0.002) since their frequency decreased in the MMI monotherapy group (-0.7%, CI 95% -1.5; 0.1%) and increased in the intervention group (1.1%, CI 95% 0.4; 1.7%) (**Table 2**).

**Table 2:** Temporal changes of natural killer and T regulatory cells percentages from baseline to 180 days.

	MMI		MMI+ViD+Se		p*	p**
	mean	Δ (CI 95%)	mean	Δ (CI 95%)		
Total NK, %						
*baseline*	11	–	17.2	–		0.10
*180 days*	7.4	-3.6 (-9; 1.8)	6.9	-10.3 (-15.8; -4.8)	0.09	0.69
CD56^bright^NK, %						
*baseline*	13.4	–	16.4	–		0.37
*180 days*	12.5	-0.9 (-4.4; 2.6)	9.9	-6.5 (-10.1; -3)	**0.03**	0.31
CD56^dim^NK, %						
*baseline*	82.1	–	80.5	–		0.70
*180 days*	85.3	3.2 (-1; 7.4)	87.4	6.9 (2.6; 11.1)	0.23	0.44
Treg, %						
*baseline*	7.7	–	7.3	–		0.55
*180 days*	7	-0.7 (-1.5; 0.1)	8.3	1.1 (0.4; 1.7)	**0.002**	0.09

NK, Natural Killer cells; Treg, T regulatory cells; p*=interaction between time course and treatment group; p**=intergroup comparison of mean values at different temporal points (baseline and after 180 days); %, frequency. Δ (IC 95%): temporal changes from baseline (confidence interval 95%). Bold values idientified p<0.05. Bold values for p values means statistically significant difference.

## Discussion

We recently showed that the association of Se and VitD to MMI, as compared to MMI alone, resulted in a significantly greater improvement of hyperthyroidism and quality of life score (Thyroid-Related Quality of Life Measure questionnaire) in patients with newly onset GD who had low Se and VitD concentrations (11). In this study, in the same group of GD patients, we investigated whether the combination treatment had immunomodulatory effects that may have contributed to the greater improvement of thyroid function.

In our study, GD patients had significantly higher frequencies of total and CD56^bright^NK cells than controls. NK cells, and particularly CD56^bright^ subset, are expected to be involved in autoimmunity due to their ability to distinguish between transformed/stressed cells and healthy cells, to orchestrate both innate and adaptive immune cells and to interact with stromal cells, through the integration of signals from activating (such as CD69, NKG2D) and inhibitory (such as CD161) receptors. The enlargement of NK cells expressing CD69, which is a marker of precocious activation might reflect the unsuccessful attempt to control inflammation at the very beginning. However, degranulation capability was significantly impaired compared to HC. In keeping with our results, Solerte et al. reported depressed NK cells cytotoxicity and reduced release of IL-2 and TNF-α in a small sample of GD patients compared to HC (21, 22). This might be a consequence of the detrimental effect of hyperthyroidism (21), since a decreased secretion of cytolytic granules was observed in NK cells of thyrotoxic mice (due to levothyroxine treatment) compared to euthyroid mice. From a bidirectional perspective, abnormal thyroid hormone levels negatively impact on the immune response, and conversely, immune system dysfunction may promote the onset/perpetuation of immune-mediated thyroid dysfunction. In this scenario, impaired degranulation might have enabled the aberrant expansion of pro-inflammatory immune subsets, indirectly supporting the escalation of the auto-inflammatory process (23, 24). Laroni et al. have shown that CD56^bright^CD16^low^NK cells subset was able to inhibit autologous CD4^+^T cell proliferation (25), which was associated with multiple sclerosis course. Another notable feature of CD56^bright^CD16^low^ NK cell function was that these cells are capable of inhibiting CD4^+^T proliferation through the release of adenosine (26), an important immunosuppressive molecule with potentially relevant roles in autoimmune diseases (27). In the present study, during treatment, NK cell percentage, and especially CD69^+^CD56^bright^NK cells decreased in both groups of treatment, but more consistently in the intervention group, which exhibited a significantly greater and prompter recovery from hyperthyroidism. Likely, NK cell percentage followed the course of the disease with a protective role. Thyrocytes may produce warning signals to attract immune cells after exposure to stressful conditions (such as stress, a viral infection, or smoking) and would theoretically switch off these signals when thyroid function improves (28). The anti-inflammatory and protective (29) effect of micronutrient supplementation on thyroid microenvironment might have supported the greater reduction of activated/dysregulated NK cells in the supplemented group. Treg cell percentage increased throughout therapy exclusively in the intervention group, likely facilitating the clinical remission of GD (30). Interestingly, 161^+^NK cells frequency was inversely related to baseline VitD levels. In mice, oral VitD supplementation increased FoxP3^+^Treg cell percentage (13, 31, 32), decreased pro-inflammatory T helper 17 infiltration and reduced the synthesis of inflammatory cytokines by T helper cells (13, 33). Similarly, oral Se supplementation stimulated Treg cell percentage, decreased thyroid immune infiltration and circulating thyroid antibodies in AITD mice (34). Thus, optimization of Se and ViD status might have quenched inflammation and supported the prompter remission of the disease. Further functional and *in vitro* studies on immune subsets and immunohistochemistry experiments have been scheduled to elucidate these findings.

As far as we know, this is the first study exploring the combined effect of Se and VitD supplementation in GD management. The major strength is that the study was designed as a randomized clinical trial. Thus, the effect of the combined supplementation was directly compared to standard treatment. Other strengths included strict enrolment criteria and VitD and Se status tests before enrollment. Due to the large number of cases required to obtain an adequate sample size, a factorial study randomizing patients in four arms (Se+placebo+MMI vs. placebo+VitD+MMI vs. Se+VitD+MMI vs. MMI alone) was not feasible in our single Centre. Therefore, it was not possible to specify the contribution of Se and VitD supplementation on their own. Another limitation was that the two randomized groups were not balanced in terms of GD severity and VitD levels at baseline due to the premature interruption of recruitment due to the SARS-CoV-2 pandemic spread. To partially overcome this potential bias, baseline FT4 and VitD levels were included as a covariate in statistical analysis. Additionally, to achieve a similar final VitD status, a different initial bolus dose of cholecalciferol was established in accordance with baseline VitD levels. We acknowledge that a different VitD status at baseline (although all patients had VitD levels lower than 30 ng/ml) might have influenced the immune subset. Actually, no significant correlations emerged between VitD levels, NK cell and Treg cell frequency, but lower VitD levels were correlated to a higher frequency of NK cells expressing the inhibitory marker CD161. Thus, this issue should be considered as a possible bias. Since NK cell degranulation and phenotype were highly modulated in GD and HC, sample size extension of both GD and HC and longer follow-up of patients are needed.

In conclusion, the results of this study in a cohort of newly diagnosed, consecutive GD patients, with borderline low Se levels and VitD insufficiency, suggest that Se and VitD supplementation may contribute to the restoration of immune homeostasis during MMI treatment. It is reasonable to propose the assessment of Se and VitD status at diagnosis of GD, and supplementation at adequate and safe dosages in case of deficiency of these micronutrients.

## Data availability statement

The raw data supporting the conclusions of this article will be made available by the authors, without undue reservation and upon specific request, by the corresponding author.

## Ethics statement

The studies involving human participants were reviewed and approved by the Local Ethic Commity, registration number 92/2017. Registration number on Eudract Clinical Trial Register SeMMIviD2017-005050-11. The patients/participants provided their written informed consent to participate in this study.

## Author contributions

DG, EP, LB, GV, LM, MLT and AP designed the study protocol. LM, AB, MG, SAMC, and AG designed and performed immune tests. DG, AB, MG, SAMC, GV and LM analyzed the data and performed the statistical analysis. DG, MG, AB, LM and GV prepared tables and figures. DG, EP, LB, and MLT were responsible for clinical management. AB, EP, and LM provided funds. All the Authors contributed to manuscript drafting and approved the submitted version of the manuscript.

## Supplementary material

Supplementary Table 1 Main demographic and immunological features of Graves’ disease patients and sex and age healthy controls.

Supplementary Table 2 Correlation between vitamin D, selenium, T regulatory cells, NK cells (total and NK cells subsets) and age.
