# Supplementary material for: Immunomodulatory role of vitamin D and selenium supplementation in newly diagnosed Graves’ disease patients during methimazole treatment
Source: Front Endocrinol (Lausanne). 2023 Apr 14;14:1145811. doi: 10.3389/fendo.2023.1145811 (PMC10141462; doi:10.3389/fendo.2023.1145811)
Supplement: Supplementary Table 1 — Main demographic and immunological features of Graves’ disease patients and sex and age healthy controls. [file Table_1.pdf]

Supplementary Table 1. Main demographic and immunological features of Graves' disease patients and sex and age healthy controls.

Parameters	***GD** (n=42)*	***HC** (n=75)*	***p-value***
***age**, years*	46.7±10.3	43.7±10.4	0.14
***men**, n (%)*	5 (12.2%)	18 (24.0%)	0.13
***NK**, %*	15.7±9.6	9.9±5.6	**0.001**
***CD56 ^{bright}NK**, %*	12.2±10.3	7.3±4.1	**0.02**
***CD56^{dim}NK**, %*	84.5±11.5	88.1±5.8	0.10
***Treg**, %*	7.4±2	6.8±1.7	0.11

Data were reported as mean ± standard deviation, or number (percentage). p-value = testing the hypothesis of null difference between groups (T test); in **bold** if <0.05.

Abbreviations: GD, Graves' disease patients; HC, healthy controls; NK, total circulating natural Killer cells expressed as the percentage of $CD3^-$cells; Treg, T regulatory cells expressed as a percentage of $CD4^+$ cells.
