# Supplementary material for: Immunomodulatory role of vitamin D and selenium supplementation in newly diagnosed Graves’ disease patients during methimazole treatment
Source: Front Endocrinol (Lausanne). 2023 Apr 14;14:1145811. doi: 10.3389/fendo.2023.1145811 (PMC10141462; doi:10.3389/fendo.2023.1145811)
Supplement: Supplementary Table 2 — Correlation between vitamin D, selenium, T regulatory cells, NK cells (total and NK cells subsets) and age. [file Table_2.pdf]

Supplementary Table 2: Correlation between vitamin D, selenium, T regulatory cells, NK cells (total and subsets) and age.

	VitD	*Se*	*Treg*	*NK*	*$CD56^{bright}NK$*	*$CD56^{dim}NK$*	*Age*
VitD, ng/ml	1	0.034	0.219	-0.072	-0.157	0.114	0.211
Se, mcg/l		1	0.161	-0.136	-0.216	0.170	0.000
Treg, %			1	0.124	-0.334	0.352	**0.552**
NK, %				1	0.048	-0.036	0.327
$CD56^{bright}NK$, %					1	**-0.979**	**-0.362**
$CD56^{dim}NK$, %						1	0.319
Age, years							1

Pearson correlation test. Bold: $p<0.005$. High correlation for value near 1: negative (-), positive (+).
